# The M235T Polymorphism in the *AGT* Gene and CHD Risk: Evidence of a Hardy-Weinberg Equilibrium Violation and Publication Bias in a Meta-Analysis

**DOI:** 10.1371/journal.pone.0002533

**Published:** 2008-06-25

**Authors:** Mohammad Hadi Zafarmand, Yvonne T. van der Schouw, Diederick E. Grobbee, Peter W. de Leeuw, Michiel L. Bots

**Affiliations:** 1 Julius Center for Health Sciences and Primary Care, University Medical Center Utrecht, Utrecht, The Netherlands; 2 Persian Gulf Health Research Center, Bushehr University of Medical Sciences and Health Services, Bushehr, Iran; 3 Department of Internal Medicine, University Hospital Maastricht, Maastricht, The Netherlands; Innsbruck Medical University, Austria

## Abstract

**Background:**

The M235T polymorphism in the *AGT* gene has been related to an increased risk of hypertension. This finding may also suggest an increased risk of coronary heart disease (CHD).

**Methodology/Principal Findings:**

A case-cohort study was conducted in 1,732 unrelated middle-age women (210 CHD cases and 1,522 controls) from a prospective cohort of 15,236 initially healthy Dutch women. We applied a Cox proportional hazards model to study the association of the polymorphism with acute myocardial infarction (AMI) (n = 71) and CHD. In the case-cohort study, no increased risk for CHD was found under the additive genetic model (hazard ratio [HR] = 1.20; 95% confidence interval [CI], 0.86 to 1.68; *P* = *0.28*). This result was not changed by adjustment (HR = 1.17; 95% CI, 0.83 to 1.64; *P* = *0.38*) nor by using dominant, recessive and pairwise genetic models. Analyses for AMI risk under the additive genetic model also did not show any statistically significant association (crude HR = 1.14; 95% CI, 0.93 to 1.39; *P* = *0.20*). To evaluate the association, a comprehensive systematic review and meta-analysis were undertaken of all studies published up to February 2007 (searched through PubMed/MEDLINE, Web of Science and EMBASE). The meta-analysis (38 studies with 13284 cases and 18722 controls) showed a per-allele odds ratio (OR) of 1.08 (95% CI, 1.01 to 1.15; *P* = *0.02*). Moderate to large levels of heterogeneity were identified between studies. Hardy-Weinberg equilibrium (HWE) violation and the mean age of cases were statistically significant sources of the observed variation. In a stratum of non-HWE violation studies, there was no effect. An asymmetric funnel plot, the Egger's test (*P* = 0.066), and the Begg-Mazumdar test (*P* = 0.074) were all suggestive of the presence of publication bias.

**Conclusions/Significance:**

The pooled OR of the present meta-analysis, including our own data, presented evidence that there is an increase in the risk of CHD conferred by the M235T variant of the *AGT* gene. However, the relevance of this weakly positive overall association remains uncertain because it may be due to various residual biases, including HWE-violation and publication biases.

## Introduction

Angiotensinogen (AGT) is a liver protein that interacts with renin to produce angiotensin I, the pro-hormone of angiotensin II. Angiotensin II is the major effector molecule of the renin-angiotensin-aldosterone system (RAAS) and plays a key role in the regulation of blood pressure (BP) by increasing vascular tone and promoting sodium retention. Genetic variants in the angiotensinogen gene modify the plasma concentration of angiotensinogen, which has been directly related to arterial blood pressure [1]. The molecular variant (M235T) of the *AGT* gene, encoding a threonine instead of a methionine at residue 235 of the mature protein, has been associated with a higher plasma AGT level and higher BP in patients homozygous for the T allele and occurs among various ethnic populations [1]–[3]. In a meta-analysis, the TT genotype was associated with a 32% increase in the risk of hypertension in white people but not in non-white people, when compared with the MM genotype [4].

Given the importance of hypertension in the occurrence of coronary heart disease [5], this finding suggests that this polymorphism may be related to increased risk of CHD. A few studies [6]–[8], including recent publications, [9], [10] have found that there is an association of the M235T *AGT* variant with increased CHD risk; however, this relationship was not confirmed in several other studies [11]–[13] as well as in a meta-analysis [14]. Marked ethnic differences in the frequency of the T allele, small sample sizes and genotyping or phenotyping errors could partly account for discrepancies among these gene-disease association studies. Therefore, we investigated the association of the M235T polymorphism in the *AGT* gene (National Center for Biotechnology Information single nucleotide polymorphism cluster ID rs699) with acute myocardial infarction (AMI) and CHD in a large population-based cohort of middle-aged Dutch women and conducted an updated meta-analysis of the available studies to clarify the role of the M235T polymorphism in CHD risk.

## Methods

### Case-cohort study

Study design, general questionnaire, anthropometric and laboratory measurements have been described in detail elsewhere [15]–[16]. Briefly, the study population consisted of participants of the Prospect-EPIC cohort. Participants were recruited between 1993 and 1997 among women living in Utrecht and the vicinity who attended the regional population-based breast cancer-screening program. A total of 17,357 women, aged 49–70, were included. At baseline, a general and a dietary questionnaire were administered, a limited physical examination was performed and a non-fasting blood sample was taken. Follow-up event information was obtained from the Dutch Centre for Health Care Information, which holds a standardized computerized register of hospital discharge diagnoses. Using the International Classification of Diseases, ninth Revision (ICD-9) codes for the main discharge reason, we categorized cardiovascular disease (codes 390–459) as CHD (codes 410–414), including AMI (code 410), and other cardiovascular diseases. Whenever multiple events (AMI and CHD) occurred, the first occurrence of that endpoint was taken as the endpoint of interest in endpoint-specific analyses. All women signed an informed consent form prior to study inclusion. The study was approved by the Institutional Review Board of the University Medical Center Utrecht.

We applied the case-cohort design introduced by Prentice [17]. From the 17,357 women in the total cohort, we randomly selected a sample of 10% as the sub-cohort (n = l736). Women who did not consent to linkage with vital status registries or who were not traceable (cases n = 3/sub-cohort n = 38) were not included. Women who reported a diagnosis of cardiovascular disease (ICD-9; 390–459) at baseline or who had missing questionnaires, blood, or DNA samples were excluded. This resulted in 15,236 women in the total cohort and 1522 women in the sub-cohort (as the control group) at baseline. All individuals with first fatal and non-fatal CHD and ischemic stroke events that arose during follow-up until January 1^st^ 2000 were selected as cases. These were 211 CHD cases, including 71 AMIs. For all case subjects, follow-up ended at the date of diagnosis or at the date of death due to cardiovascular disease.

#### Genetic analysis

Genetic analysis was performed at the Cardiovascular Genotyping (CAGT) laboratory of the Department of Internal Medicine of the University Hospital Maastricht. Genomic DNA was extracted from buffy coats using the QIAamp® Blood Kit (Qiagen Inc., Valencia, California, USA). Genotyping of the polymorphisms was performed using a multilocus genotyping assay for candidate markers of cardiovascular disease risk (Roche Molecular Systems Inc., Pleasanton, CA, USA) [18]. Briefly, each DNA sample was amplified using two multiplex polymerase chain reactions, and the alleles were genotyped simultaneously using an array of immobilized sequence-specific oligonucleotide probes. This array of probes was blotted on plastic strips, and, after staining, genotypes were scored based on blue (positive) and white (negative) bands. Each blue band, representing a specific genotype, was scored by specific software (counting the pixel intensity of each band) and checked manually. Genotyping was performed blinded to the case-control status. A random double-check was performed to detect potential genotyping errors in a subset of 100 samples. The check confirmed the previous genotyping results by 100%.

#### Data analysis

Hardy-Weinberg equilibrium (HWE) was tested with the χ^2^ test among the controls. Allele frequencies were estimated by gene counting. We used the ANOVA *F* test to estimate relationships among the M235T genotypes and continuous variables, while we tested the significance of any difference in proportions by applying the χ^2^ statistic. A p-value <0.05 (2-sided) was considered statistically significant.

To assess the relationship of the M235T polymorphism in the *AGT* gene with the outcome, we used a Cox proportional hazards model with an estimation procedure adapted for case-cohort designs. We used the unweighted method by Prentice [17], [19], which is incorporated in a SAS macro at http://lib.stat.cmu.edu/general/robphreg. A previous meta-analysis [14] showed that the effect of the *AGT* M235T variant on its intermediate phenotype (plasma angiotensinogen level) follows an additive model according to the number of T alleles [5% (95% CI: 2 to 8%) increase for the MT and 11% (95% CI: 7 to 15%) increase for the TT genotype versus the MM genotype]. Therefore, our *priori* hypothesis was that the association between the M235T polymorphism in the *AGT* gene and CHD follows an additive model according to the number of T alleles. However, other genetic models were evaluated as well. We considered different modes of inheritance as follows: *the additive “per-allele” model*, the T allele was compared between cases and controls by assigning scores of 0, 1, and 2 to homozygotes for the M allele, heterozygotes, and homozygotes for the T allele, respectively; *the recessive model*, the TT genotype versus the MT and MM combined genotypes; and *the dominant model*, the MT and TT genotypes combined versus the MM genotype. We also performed separate pairwise comparisons of the MT and TT genotypes versus the MM genotype.

### Meta-analysis

#### Searching

We searched PubMed/MEDLINE, Web of Science, and EMBASE up to February 2007 for observational studies evaluating an association between the M235T polymorphism in the *AGT* gene and CHD. Terms used for the search contained both medical subject heading terms and text words: (Met235Thr OR M235T OR T704C) AND (angiotensinogen OR *AGT*) AND (polymorphism OR mutation OR genetic OR genotype) AND (“coronary disease” OR “coronary heart disease” OR CHD OR “myocardial infarction” OR MI OR “myocardial infarct” OR “coronary artery disease” OR CAD OR “ischemic heart disease” OR IHD OR “cardiovascular disease” OR “heart disease” OR angina). We also retrieved additional studies by hand searching the bibliographies of original research reports and review articles and through the MEDLINE option “related articles”. Search results were limited to articles published in English and studies on human subjects.

#### Selection

All studies were considered potentially eligible if they aimed to investigate the relationship between the M235T genotypes and risk of CHD or MI. Any observational study, regardless of sample size, which fulfilled the following criteria, was included: (i) *AGT* M235T genotype frequencies were provided by case-control status (studies without controls were excluded); (ii) risk of CHD or MI was evaluated (studies on recurrent coronary events were excluded); (iii) relevant data were presented to calculate the effect size and its 95% CI; (iv) non-overlapping data were contained. For duplicate publications, the study with the smaller data set was excluded.

#### Data abstraction

The following information was extracted from each study that we included: the first author's name; country; year of publication; the population evaluated; study design; mean age or age range for case-patients and controls; definition and number of cases and controls; allele frequencies and genotype distribution in case-patients and controls (where data were not given, they were calculated from the corresponding genotyping frequencies of the case and control groups); consistency of genotype frequencies with HWE (calculated); gender in the evaluated population and male percentage, matching variables, use of blinding of genotyping staff, performing regenotyping of a random sample, and crude ORs and 95% CIs for development of CHD or MI related to the *AGT* gene genotypes based on different genetic models (from the original paper or calculated from crude data if not provided). We again considered a dominant, a recessive, an additive “per-allele” model and pairwise comparisons. Data were extracted independently and entered into separate databases by two authors (performed by MHZ and MLB). Results were compared, and disagreements were resolved by a consensus.

#### Quantitative data synthesis

The method of Mantel-Haenszel was used to calculate the odds ratio for the pooled data in a fixed-effects model, and, if there was evidence for heterogeneity, the DerSimonian-Laird method was used for the pooled odds ratio in a random-effects model, under pairwise comparisons of the different genotypes and dominant, recessive, and additive inheritance models. For all the models used, the T allele was considered the risk allele. The genetic model to be considered as the *priori* hypothesis was the additive model. In each study, we tested for HWE by using the χ^2^ test or an exact test among the controls by using the genhwi command in Stata 9.2 [20].

In addition, we used Cochran's χ^2^ – based ***Q*** statistic for between-study heterogeneity, which is considered to be significant for *P*<0.10, as well as the ***I^2^*** statistic for estimation of inconsistency in meta-analyses [20]. ***I^2^*** represents the percentage of the observed between-study variability due to heterogeneity rather than to chance. It ranges between 0% and 100%, where a value of 0% indicates no observed heterogeneity, and larger values indicate an increasing degree of heterogeneity (roughly suggested cut-off points include: ***I^2^*** = 0–25%, no heterogeneity; ***I^2^*** = 25–50%, moderate heterogeneity; ***I^2^*** = 50–75%, large heterogeneity; ***I^2^*** = 75–100%, extreme heterogeneity) [21].

We used funnel plots to examine the publication bias of reported associations. We also used Egger's test and the Begg-Mazumdar test with 95% CI for evaluation of publication bias, which are considered to be significant for *P*<0.10. Meta-analysis was carried out using STATA 9.2. We used random effect meta-regression models with restricted maximum likelihood estimation to evaluate the extent to which different variables explained heterogeneity among the individual ORs. The pre-specified characteristics for assessment of sources of inter-study heterogeneity were: study size (for detailed definition see [22]); ethnicity of population evaluated (of Caucasian descent, East Asian, and others); male percentage in each study, matching (matched or unmatched); blinding of genotyping staff (blinded, or not reported); performing regenotyping of a random sample (performed or not reported); violating HWE (violated or confirmed; the term “violated” used for statistically significant deviation of HWE) in sub-group analysis as well as in meta-regression analysis.

#### HWE Correction

For evaluating the impact of HWE-violated studies on effect estimates (at the 0.05 significance level) under different genetic models, odds ratios, and variances were corrected by using the HWE-predicted genotype counts in the control instead of the observed counts as previously suggested [20]. Thereafter, they were included in the sensitivity analysis.

## Results

### Prospect-EPIC study results

The general characteristics of the randomly sampled participants of the cohort (N = 1522) are given in Table 1. The genotype distribution was in Hardy-Weinberg equilibrium (χ^2^ = *0.020*; *P* = *0.89*). General and clinical characteristics of CHD cases and controls are shown in Table 1. The median follow up time for the random sample was 4.3 years, with a total of 6,523 person years. The actual follow-up in the baseline cohort of 15,236 women was 64,768 person years. Due to the case-cohort design, 23 women in the sub-cohort eventually were CHD cases (among which there were nine AMI cases).

**Table 1:** Baseline characteristics of the sub-cohort according to genotype, and clinical characteristics of CHD cases and controls in the Prospect –Epic cohort.

Characteristics		sub-cohort (N = 1522)			*P*-value^b^	CHD cases	Sub-cohort	*P*-value^c^
		M235M	M235T	T235T				
N total (%)		535 (35.2)	737 (48.4)	250 (16.4)	-	210	1522	-
Age at intake (yr) a		57.1±5.8	57.1±6.2	57.4±6.3	0.83	60.5±5.9	57.1±6.1	<0.01
Body mass index (kg/m^2^) a		26.0±4.1	25.6±3.8	25.8±4.1	0.19	26.8±3.9	25.8±4.0	<0.01
Weight (kg) a		70±11	69±11	69±11	0.17	71±11	69±11	0.07
Height (cm) a		164.4±5.9	164.2±6.0	164.0±6.1	0.66	162.8±6.0	164.3±6.0	<0.01
Waist to hip ratio a		0.794±0.057	0.786±0.058	0.786±0.055	0.03	0.813±0.060	0.789±0.057	<0.01
Hypertension (%) d		39.4	41.2	48.4	0.06	60.5	41.8	<0.01
Systolic blood pressure (mm Hg) a		131±19	133±21	135±20	0.07	143±22	133±20	<0.01
Diastolic blood pressure (mm Hg) a		79±10	79±11	80±11	0.14	82±11	79±11	<0.01
Presence of diabetes (%)		2.2	2.0	2.8	0.78	5.7	2.2	<0.01
Presence of hypercholesterolemia (%)		3.6	4.6	2.8	0.38	11.4	3.9	<0.01
Current alcohol consumption (%)		88.7	87.1	89.2	0.60	80.7	88.0	<0.01
Smoking status (%)	Past	35.1	33.8	36.4	0.73	26.2	34.7	0.02
	Current	23.2	22.4	23.6	0.90	33.8	22.9	<0.01
Pack- years e		6.8±9.5	6.5±9.5	6.7±9.3	0.87	9.7±11.4	6.7±9.5	<0.01
Total cholesterol (mmol/L) a		5.9±1.0	5.8±0.9	5.9±1.1	0.05	6.4±1.0	5.9±1.0	<0.01
HDL cholesterol (mmol/L) a		1.6±0.4	1.6±0.4	1.6±0.4	0.33	1.4±0.3	1.6±0.4	<0.01
LDL cholesterol (mmol/L) a		4.0±1.0	3.9±0.9	3.9±0.9	0.25	4.4±1.0	3.9±0.9	<0.01
Serum glucose (mmol/L) a		4.6±1.5	4.5±1.3	4.5±1.2	0.52	5.1±2.5	4.5±1.4	<0.01

HDL, high-density lipoprotein; LDL, low-density lipoprotein; CHD, coronary heart disease (ICD 410–414).

a Mean±standard deviation.

b Comparison of risk factors across genotypes, using the ANOVA *F* test (continuous variables) and the χ^2^ statistic (categorical variables).

c Comparison of risk factors across disease status, using the *independent samples t-test* (continuous variables) and the χ^2^ statistic (categorical variables).

d Defined as a systolic blood pressure ≥140 mm Hg and/or diastolic blood pressure ≥90 mm Hg and/or questionnaire positive.

e The number of packs of cigarettes smoked per day by the number of years the person has smoked.

Due to the association of the M235T genotypes with some risk factors of CHD, we presented crude models and models adjusted for hypertension, total cholesterol and waist to hip ratio as potential confounding factors. Table 2 presents hazard ratios of AMI and CHD under different genetic models. Under the additive model of inheritance, no increased risk for CHD was found (HR = 1.20; 95% CI, 0.86 to 1.68; *P* = *0.28*), which did not alter after adjustment (HR = 1.17; 95% CI, 0.83 to 1.64; *P* = *0.38*). The same was true for other comparisons (Table 2). Analyses for AMI risk did not show any statistically significant associations (Table 2).

**Table 2:** Association of the *AGT* M235T polymorphism and AMI and CHD under different genetic models.

Mode of Inheritance	Crude: model 1			Adjusted: model 2 b		
	Hazard ratio	95% CI	P-value	Hazard ratio	95% CI	P-value
**AMI**						
Additive a	1.20	0.86–1.68	0.28	1.17	0.83–1.64	0.38
Recessive (TT vs. M-carriers)	0.77	0.43–1.41	0.40	0.87	0.46–1.58	0.62
Dominant (T-carriers vs. MM)	0.79	0.47–1.32	0.36	0.79	0.46–1.33	0.37
MT vs. MM	1.09	0.84–1.41	0.53	1.11	0. 85–1.45	0.45
TT vs. MM	1.21	0.86–1.70	0.28	1.17	0.83–1.63	0.38
**CHD**						
Additive a	1.14	0.93–1.39	0.20	1.11	0.90–1.38	0.33
Recessive (TT vs. M-carriers)	0.87	0.60–1.26	0.45	0.98	0.66–1.47	0.93
Dominant (T-carriers vs. MM)	0.82	0.60–1.12	0.21	0.80	0.58–1.10	0.18
MT vs. MM	1.09	0.93–1.27	0.31	1.13	0.95–1.34	0.16
TT vs. MM	1.14	0.93–1.40	0.20	1.11	0.90–1.37	0.33

AMI = acute myocardial infarction (ICD 410); CHD = coronary heart disease (ICD 410–414).

a The additive genetic model assumes that there is a linear gradient in risk between the MM, MT and TT genotypes (MM genotype baseline). This is equivalent to a comparison of the T allele versus the M allele (baseline).

b We used a cox proportional hazards model with an estimation procedure adapted for case-cohort designs; adjusted for waist to hip ratio, hypertension, total cholesterol.

### Meta-Analysis results

**Figure 1:**
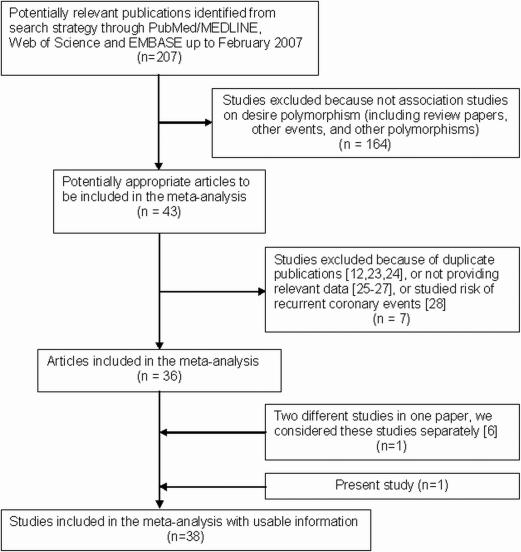
Flow chart of study selection.

***Flow of included studies***. A total of 44 gene-disease association studies, including the present study, evaluating the *AGT* M235T gene variant and CHD risk were identified. Seven articles were excluded, three of which were duplicate publications [12], [23], [24], three of which did not provide relevant data [25]–[27], and one of which studied the risk of recurrent coronary events [28]. Finally, 37 studies met the selection criteria. In one paper, the provided results were based on two different studies [6], so both were included in the meta-analysis. Therefore, 38 studies with 13,284 cases and 18,722 controls were included in the final meta-analysis (Figure 1).

### Study characteristics

Characteristics of the studies are shown in Table 3 [6]–[8], [10], [11], [13], [29]–[58]. There were 25 studies in Caucasians, eight studies in East Asians, and five studies in other populations (West Asian, South Asian, African, African-American, and South American). The last was collapsed into a miscellaneous group. The design of the studies was case-control, except for three studies that were prospective cohort [56], case-cohort (present study), and cross-sectional [40]. The T allele frequency varied from 26 to 54 percent in Caucasians, 65 to 91 percent in East Asians, and 34 to 83 percent in the miscellaneous group.

**Table 3:** Characteristics of published studies of the association between the M235T polymorphism in *AGT* gene and CHD included in the meta-analysis.

	Author	Year	Country	Ethnicity	Total cases	Total controls	Study size based on average weight	Cases MM	Cases MT	Cases TT	Controls MM	Controls MT	Controls TT
1	Katsuya et al. [45]	1995	New Zealand	Caucasian	422	406	Large	144	186	92	156	191	59
2	Tiret et al. [13]	1995	France and UK	Caucasian	630	741	Large	229	301	100	258	372	111
3	Ludwig et al.^a^ [6] (Framingham study)	1997	USA	Caucasian	58	55	Small	17	30	11	20	23	12
4	Ludwig et al.^b^ [6] (ARIC study)	1997	USA	Caucasian	255	245	Large	79	117	59	85	118	42
5	Wenzel et al. [55]	1997	Germany	Caucasian	111	102	Small	25	59	27	39	46	17
6	Winkelmann et al. [57]	1999	Germany	Caucasian	329	92	Small	103	148	78	28	53	11
7	Fernandez-Arcas et al. [37]	1999	Spain	Caucasian	272	182	Small	84	132	56	36	96	50
8	Gardemann et al. [40]	1999	Germany	Caucasian	1739	511	Large	536	920	283	168	247	96
9	Fatini et al. [36]	2000	Italy	Caucasian	205	209	Small	61	91	53	84	86	39
10	Fomicheva et al. [38]	2000	Russia	Caucasian	198	152	Small	63	85	50	43	75	34
11	Reinhardt et al. [50]	2000	Germany	Caucasian	184	155	Small	56	101	27	38	91	26
12	Batalla et al. [31]	2000	Spain	Caucasian	220	200	Small	69	99	52	64	96	40
13	Wierzbicki et al. [56]	2000	UK	Caucasian	48	108	Small	23	21	4	58	44	6
14	Rodriguez-Perez et al. [8]	2001	Spain	Caucasian	299	315	Large	67	145	87	97	158	60
15	Olivieri et al. [7]	2001	Italy	Caucasian	454	245	Large	148	205	101	74	114	57
16	Sethi et al. [29]	2001	Denmark	Caucasian	943	7975	Large	335	460	148	2779	3886	1310
17	Ortlepp et al. [48]	2002	Germany	Caucasian	100	100	Small	25	58	17	29	55	16
18	Ermis et al. [35]	2002	Turkey	Caucasian	102	114	Small	32	48	22	39	59	16
19	Bis et al. [32]	2003	USA	Caucasian	208	717	Large	71	98	39	215	349	153
20	Buraczynska et al. [33]	2003	Poland	Caucasian	200	200	Small	28	122	50	72	80	48
21	Tobin et al. [53]	2004	UK	Caucasian	547	505	Large	212	252	83	197	226	82
22	Sekuri et al. [10]	2005	Turkey	Caucasian	115	128	Small	46	42	27	33	71	24
23	Methot et al. [47]	2005	Canada	Caucasian	198	149	Small	65	93	40	60	70	19
24	Renner et al. [51]	2005	Austria	Caucasian	2582	732	Large	841	1205	536	237	357	138
25	Zafarmand et al. (present study)	2008	Netherlands	Caucasian	210	1522	Large	64	108	38	535	737	250
26	Kamitani et al. [44]	1995	Japan	East Asian	103	103	Small	6	31	66	10	41	52
27	Ishigami et al. [43]	1995	Japan	East Asian	82	160	Small	6	22	54	30	51	79
28	Yamakawa-Kobayashi et al. [58]	1995	Japan	East Asian	315	380	Small	15	91	209	9	131	240
29	Ko et al. [46]	1997	China	East Asian	267	337	Small	6	36	225	4	54	279
30	Ichihara et al. [42]	1997	Japan	East Asian	327	352	Small	15	103	209	13	112	227
31	Cong et al. [34]	1998	Japan	East Asian	104	170	Small	2	31	71	16	43	111
32	Sheu et al. [52]	1998	China	East Asian	102	145	Small	1	26	75	1	37	107
33	Tsai et al. [54]	2006	Taiwan	East Asian	735	519	Large	15	195	525	5	111	403
34	Frossard et al. [39]	1998	UAE	Arab	74	61	Small	21	32	21	16	26	19
35	Hooper et al. [41]	2002	USA	African- American	100	100	Small	4	29	67	2	31	67
36	Nair et al. [11]	2003	India	South Asian	141	131	Small	9	36	96	11	40	80
37	Araujo et al. [30]	2004	Brazil	South American	110	104	Small	46	52	12	43	51	10
38	Ranjith et al. [49]	2004	South Africa	African	195	300	Small	24	80	91	29	127	144

Author	Study design	Mean age±SD (years) in Cases	Mean age±SD (years) in Controls	Sex	Male percent	Matching variable (s)	Allele frequency 235T (%)	*P (HWE)*	Blinding of genotyping staff	Regenotyping of random subsample
Katsuya et al. [45]	Case-control	62±7	62±7	M/F	NR	None	38	0.97	NR	NR
Tiret et al. [13]	Case-control	54±0.3	53±0.3	M	100	Age	40	0.22	NR	NR
Ludwig et al.^a^ [6] (Framingham study)	Case-control	NR	NR	M/F	86	Age and sex	43	0.28	NR	NR
Ludwig et al.^b^ [6] (ARIC study)	Case-control	NR	NR	M/F	80.5	Age and sex	41	0.92	NR	NR
Wenzel et al. [55]	Case-control	42*	38*	M/F	88	None	39	0.59	NR	NR
Winkelmann et al. [57]	Case-control	56±10	56±10	M	100	None	41	0.06	NR	NR
Fernandez-Arcas et al. [37]	Case-control	67±7	60±10	M/F	42	None	54	0.41	NR	Yes
Gardemann et al. [40]	Cross-sectional	63±9	59±11	M	100	None	43	0.76	NR	NR
Fatini et al. [36]	Case-control	59±5	51±6	M/F	76	None	39	0.0476	NR	NR
Fomicheva et al. [38]	Case-control	67	11	M	100	None	47	0.90	NR	NR
Reinhardt et al. [50]	Case-control	57±11	56±14	M/F	62	None	46	0.0240	Yes	Yes
Batalla et al. [31]	Case-control	43±5	42±6	M	100	Age and ethnicity	44	0.71	NR	NR
Wierzbicki et al. [56]	Cohort	57±13	53±12	M/F	68	None	26	0.53	Yes	Yes
Rodriguez-Perez et al. [8]	Case-control	56±10	54±10	M/F	76	None	44	0.76	Yes	NR
Olivieri et al. [7]	Case-control	60±9	58±13	M/F	83.5	None	47	0.31	NR	NR
Sethi et al. [29]	Case-control	59±9	56±15	M/F	74	None	41	0.43	NR	NR
Ortlepp et al. [48]	Case-control	58±13	59±11	M/F	68	Age, sex, and prevalence of standard cardiac risk factors	43	0.23	NR	NR
Ermis et al. [35]	Case-control	42±12	40±13	NR	NR	None	40	0.40	NR	NR
Bis et al. [32]	Case-control	70	64	M/F	61.5	Age, sex and calendar year of identification	46	0.61	Yes	NR
Buraczynska et al. [33]	Case-control	53±7	47±11	M	100	None	44	0.0077	NR	NR
Tobin et al. [53]	Case-control	62±9	57±11	M/F	68	None	39	0.21	NR	Yes
Sekuri et al. [10]	Case-control	48±8	44±7	M/F	77.4	None	47	0.19	NR	NR
Methot et al. [47]	Case-control	63±10	62±7	F	0	Age	37	0.84	NR	NR
Renner et al. [51]	Case-control	64±10	58±12	M/F	74.8	None	43	0.86	Yes	NR
Zafarmand et al. (present study)	Case-cohort	61±6	57±6	F	0	None	41	0.89	Yes	Yes
Kamitani et al. [44]	Case-control	52±1	54±1	M	100	Age, sex, BMI, blood pressure, total cholesterol, smoking and history of diabetes	70	0.65	NR	NR
Ishigami et al. [43]	Case-control	62±1	60±1	M/F	68.3	None	65	0.0002	NR	NR
Yamakawa-Kobayashi et al. [58]	Case-control	57±8	51±8	M/F	80	None	80	0.07	NR	NR
Ko et al. [46]	Case-control	62±1	56±1	M/F	77	None	91	0.51*	NR	NR
Ichihara et al. [42]	Case-control	53±6	53±5	M	100	Age, sex, BMI and some CHD risk factors (history of smoking, hypertension, diabetes, hypercholesterolemia)	80	0.86	NR	NR
Cong et al. [34]	Case-control	65±1	NR	M/F	76	None	78	0.0006	NR	NR
Sheu et al. [52]	Case-control	63±1	58±1	M	100	None	87	0.47*	NR	NR
Tsai et al. [54]	Case-control	64±11	59±13	M/F	72.2	None	88	0.38	NR	NR
Frossard et al. [39]	Case-control	57±12	54±14	M/F	48.2	None	52	0.26	NR	NR
Hooper et al. [41]	Case-control	NR	NR	M/F	NR	None	83	0.73*	NR	NR
Nair et al. [11]	Case-control	56±5	48±6	M/F	82.3	Age and sex	76	0.08	NR	NR
Araujo et al. [30]	Case-control	NR	NR	M/F	66.6	None	34	0.36	NR	NR
Ranjith et al. [49]	Case-control	NR	NR	M/F	NR	Age	69	0.90	NR	NR

Author	End point	Case definition	Source of controls
Katsuya et al. [45]	CHD	Admission for treatment of myocardial infarction or unstable angina, PTCA, or CABG, or stable angina with angiographic evidence of CHD or a positive exercise test result	Controls without a history of CHD and symptoms suggesting angina from two previous studies
Tiret et al. [13]	MI	WHO MONICA category I	Electoral rolls in France and the list of general practitioners in N. Ireland
Ludwig et al.^a^ [6] (Framingham study)	CHD	Diagnosed MI by a physician, a PTCA, a CABG, prior MI in ECG, fatal CHD	Healthy controls without the conditions, no lipid-lowering medications and no family history
Ludwig et al.^b^ [6] (ARIC study)	CHD	Diagnosed MI by a physician, a percutaneous coronary angioplasty, a coronary artery bypass, prior MI, fatal CHD	Healthy controls without the conditions, no lipid-lowering medications and no family history
Wenzel et al. [55]	CHD	>50% stenosis of at least one major coronary vessel, defined as MI, PTCA, CABG	Healthy young persons without any symptoms for CVD
Winkelmann et al. [57]	CHD, MI	At least one coronary stenosis ≥ 50%	controls without coronary artery disease in coronary angiography
Fernandez-Arcas et al. [37]	MI	Typical prolonged chest pain or atypical symptoms, acute congestive heart failure, syncope, and serial cardiac enzymes elevation exceeding twice the upper limit of reference range and dynamic ECG changes typical of MI	Healthy controls with no CVD using health service identity card
Gardemann et al. [40]	CHD, MI	CHD: coronary stenosis ≥ 50% MI: Using the WHO criteria	No vessel disease in the coronary angiography
Fatini et al. [36]	CHD	History of CHD (previous MI or angina pectoris) with coronary stenosis >75% by angiography	Random healthy controls from the staff of the University
Fomicheva et al. [38]	MI	Using the WHO criteria	From secondary schools
Reinhardt et al. [50]	CHD	At least one coronary stenosis ≥ 50% of a major coronary artery with or without prior MI	Random healthy controls from the local registry office
Batalla et al. [31]	MI	WHO MONICA protocol	Healthy controls from residents of the region
Wierzbicki et al. [56]	CHD	Confirmed cardiac event, angioplasty, coronary bypass surgery, or significant lesions on angiography	No CHD
Rodriguez-Perez et al. [8]	CHD	Hospital-admitted with a diagnosis of MI or unstable angina and documented evidence of coronary artery disease by angiography	Random controls without CVD
Olivieri et al. [7]	CHD, MI	CHD: Candidate patients for CABG, having >50% stenosis of at least one major coronary vessel MI: By medical records showing diagnostic electrocardiogram and enzyme changes, and/or the typical sequelae of myocardial infarction on ventricular angiography	CHD-free group documented by angiography who were examined for other reasons in the institute
Sethi et al. [29]	CHD, MI	CHD: ICD, 8th edition, codes 410-414 MI: ICD, 8th edition, code 410	Random healthy controls without CHD, MI or CVA from the city of Copenhagen
Ortlepp et al. [48]	CHD	>50% stenosis of at least one coronary vessel	Patients without any signs of atherosclerosis in angiography
Ermis et al. [35]	Early MI	WHO criteria	Healthy subjects without a history of CHD, hypertension or diabetes
Bis et al. [32]	MI	Criteria were adapted from the Cardiovascular Health Study	Randomly selected subjects from the members of a health maintenance organization
Buraczynska et al. [33]	CHD	Hospitalized patients with unstable angina, stable angina or acute MI	Healthy subjects without family history of CHD
Tobin et al. [53]	MI	Using the WHO criteria	Healthy visitors to patients
Sekuri et al. [10]	CHD	At least one stenosis ≥ 50% in a major coronary artery or one of their branches	Healthy subjects without history of CVD
Methot et al. [47]	CHD	Acute coronary syndrome: AMI or unstable angina defined according to standard criteria	Postmenopausal women without signs or symptoms of acute or previous acute coronary syndrome
Renner et al. [51]	CHD, MI	CHD: At least one stenosis ≥ 50% in one of 15 coronary segments MI: positive history of MI or patients presented with ST elevation or non-ST elevation	Subjects without CHD (with stenoses <20%) from a cohort study
Zafarmand et al. (present study)	CHD, MI	CHD: ICD, 9th edition, codes 410-414 MI: ICD, 9th edition, code 410	Members of a 10% random sample from the whole cohort at the baseline without CVD
Kamitani et al. [44]	MI	Having MI by coronary angiography, ECG criteria, and measurements of heart-specific serum enzymes	Randomly selected subjects attending the same hospital with no CVD
Ishigami et al. [43]	CHD	At least one coronary artery with >25% luminal obstruction on average according to multiple coronary angiographic views	Hospital-admitted patients for other diseases with no CHD
Yamakawa-Kobayashi et al. [58]	CHD	At least one 75% stenosis in coronary arteries	Healthy controls
Ko et al. [46]	CHD	>50% stenosis of at least one major coronary vessel	Healthy subjects and patients without angiographic evidence of CHD
Ichihara et al. [42]	CHD	MI was based on typical ECG changes and increased serum enzymes and by the presence of wall motion abnormality on left ventriculography, Angina pectoris by typical ECG changes and stenosis of >70% in any major coronary artery or of >50% in the left main trunk, without wall motion abnormality on left ventriculography	Random healthy controls with no history or sign of CHD from attendants of the hospitals
Cong et al. [34]	CHD	≥ 50% stenosis in at least one major coronary artery	Subjects with no history of CHD or abnormal resting electrocardiogram
Sheu et al. [52]	CHD	A postnitroglycerin stenosis of major vessels ≥ 50% or a >70% reduction of luminal diameter of a first-order branche	Healthy subjects in their annual physical checkups
Tsai et al. [54]	CHD	>50% stenosis of at least one coronary vessel	CHD-free group documented by angiography
Frossard et al. [39]	CHD, MI	CHD: Exertional angina, unstable angina or MI MI: ECG changes; presence of regional wall motion abnormalities on trans-thoracic echocardiography; and serial enzyme elevations	Healthy controls
Hooper et al. [41]	MI	Prior MI confirmed by ECG and/or cardiac enzymes or cardiac thallium scanning or catheterization	Outpatients with no history of heart attack, stroke, or thrombosis
Nair et al. [11]	CHD	At least one coronary artery with 50% stenosis	Healthy controls with BP<140/90 mm Hg and no history of CVD
Araujo et al. [30]	MI	Using the WHO criteria confirmed by stenosis >50% in an angiography and ventricular damage in a ventriculography	Hospital-admitted patients for other diseases with a normal coronary angiography
Ranjith et al. [49]	MI	Using the WHO criteria	Healthy normotensive subjects with no CVD or other associated risk factors

PTCA, percutaneous coronary angioplasty; CABG, coronary artery bypass graft; ICD, international classification of diseases; ECG, electrocardiography; AMI, acute myocardial infarction; CHD, coronary heart disease; CVD, cardiovascular diseases; CVA, cerebrovascular accident; BMI, body mass index; WHO, world health organization; NR, not reported.

* Exact significance probability.

All studies used polymerase chain reaction methods for genotyping, and most used a restriction fragment length method for polymorphism analysis. Blinding of investigators involved in genotyping with respect to the case/control status of the participants was reported in six studies [8], [32], [50], [51], [56]. A random double-check to detect potential genotyping errors was mentioned in five studies [37], [50], [53], [56]. In most of the studies, the genotype frequencies were consistent with HWE. However, statistically significant deviations from HWE were found in five studies (Table 3) [33], [34], [36], [43], [50]. CHD cases were defined in 16 studies as a >50% stenosis of at least one coronary vessel [7], [8], [10], [11], [34], [40], [41], [43], [46], [48], [50], [51], [54]–[57], while, in four studies, a >70% stenosis was considered [36], [42], [52], [58]. In 14 studies [13], [29]–[32], [35], [37]–[39], [44], [47], [49], [53], the WHO criteria were used, and, in four studies, CHD was diagnosed based on a clinical diagnosis [6], [33], [45]. Controls arose from the source population of the cases in 21 studies [6], [8], [13], [29], [31]–[33], [35]–[38], [45], [47], [49]–[53], [55], while hospital-based/not population-based controls were used in 17 studies [7], [10], [11], [30], [34], [39]–[44], [46], [48], [54], [56]–[58].

### Quantitative data synthesis

**Figure 2:**
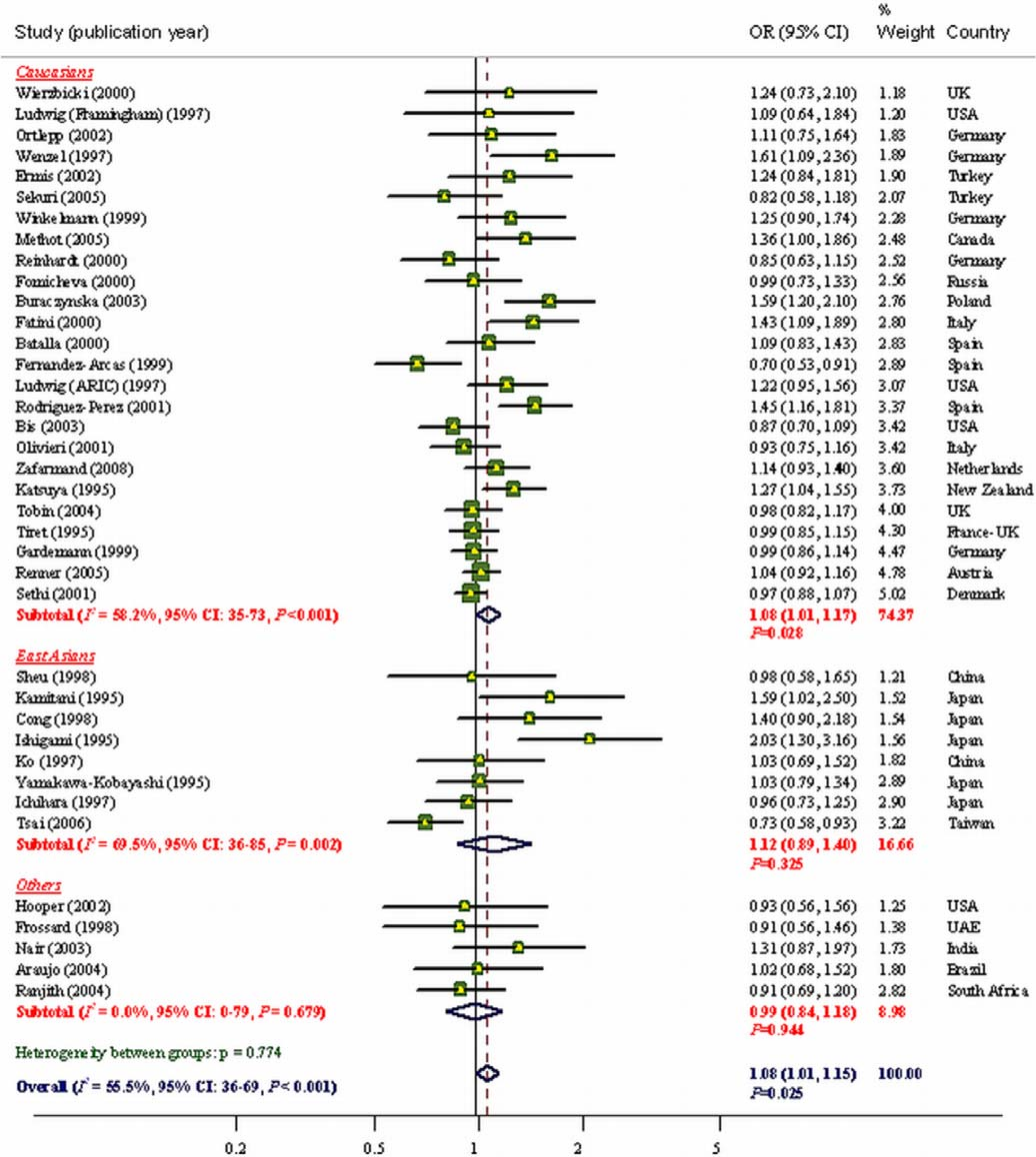
Results of published studies of association between the M235T polymorphism in *AGT* gene and coronary heart disease in different ethnic groups. ORs for the outcome compared the T235 allele vs. the M235 allele (Additive model). The size of the box is proportional to the weight of the study. Given *P*-values for odds ratios are based on DerSimonian-Laird method using a random effects model and for heterogeneity in different ethnic groups are based on Q-test.

The overall OR under a random-effects model using an additive model for CHD risk was 1.08 (95% CI, 1.01 to 1.15; *P* = 0.025; Figure 2). However, there was evidence of substantial between-study heterogeneity (***I^2^*** = 55.5%, *P*<0.001). Table 4 shows the association of the *AGT* T235M polymorphism with CHD risk under different genetic contrasts. When a recessive model was evaluated, a significant association was found between individuals homozygous for the T allele (T235T genotype) and CHD risk, when compared to carriers of the M allele (OR = 1.11; 95% CI, 1.02 to 1.22; *P* = 0.016). Under the dominant model, the association was not significant. Under pairwise comparisons, there was a significant modest association between the T235T genotype and CHD risk, as compared with the M235M genotype (OR = 1.15; 95% CI, 1.00 to 1.32; *P* = 0.045). There was evidence for moderate to large between-study heterogeneity under all models (Table 4). Sub-group analysis, by study characteristics under the additive model, showed that matching, blinding of genotyping staff, and regenotyping of a random sub-sample explained little of the heterogeneity. However, stratification showed an attenuated effect estimates in the large studies, in studies that CHD was defined based on angiography or WHO criteria, and in particular in studies that were in HWE (Table 5). Further evaluation of potential sources of the heterogeneity was performed using a meta-regression analysis.

**Table 4:** ORs and 95% CI for coronary heart disease and the M235T polymorphism in *AGT* gene under different genetic models.

Genetic model	Random effects OR (95% CI)	*P*-value	*I^2^* (%) (95% CI)	***Q*** statistic for heterogeneity (df = 37)	*P*-value for heterogeneity	Egger's test *P*-value	Begg's test *P*-value
Additive a	1.08 (1.01–1.15)	0.025	55.5 (36–69)	83.21	<0.001	0.066	0.074
Recessive (TT vs. M-carriers)	1.11 (1.02–1.22)	0.016	37.5 (7–58)	59.23	0.012	0.011	0.070
Dominant (T-carriers vs. MM)	1.07 (0.96–1.19)	0.253	56.0 (37–69)	84.02	<0.001	0.549	0.706
MT vs. MM	1.02 (0.91–1.14)	0.724	51.3 (29–66)	75.99	<0.001	0.895	0.960
TT vs. MM	1.15 (1.00–1.32)	0.045	53.3(33–68)	79.30	<0.001	0.286	0.615

a The additive genetic model assumes that there is a linear gradient in risk between the MM, MT and TT genotypes (MM genotype baseline). This is equivalent to a comparison of the T allele versus the M allele (baseline).

**Table 5:** Studies of the M235T polymorphism in *AGT* gene and risk of coronary heart disease under additive model grouped by study characteristics.

Study characteristics	Number of studies	Per-allele OR (95%CI)	*P*-value	***I^2^*** (%) (95%CI)	***Q*** statistic for heterogeneity	*P*-value for heterogeneity
Overall	38	1.08 (1.01–1.15)	0.025	55.5 (36–69)	83.21	<0.001
Study size						
Small	26	1.12 (1.02–1.24)	0.021	50.2 (35–73)	50.24	0.002
Large	12	1.03 (0.95–1.12)	0.502	62.0 (29–80)	28.92	0.002
Ethnicity						
Caucasians	25	1.08 (1.01–1.17)	0.028	58.2 (35–73)	57.43	<0.001
Eastern Asians	8	1.12 (0.89–1.40)	0.325	69.5 (36–85)	22.96	0.002
Others	5	0.99 (0.84–1.18)	0.944	0.00 (0–79)	2.31	0.679
Matching						
Matched	11	1.07 (0.96–1.18)	0.211	26.2 (0–63)	13.56	0.194
Unmatched	27	1.08 (0.99–1.17)	0.072	62.7 (44–75)	69.65	<0.001
Violating HWE						
Violated	5	1.38 (1.05,–1.83)	0.022	70.7 (26–88)	13.65	0.009
Confirmed	33	1.04 (0.98–1.11)	0.188	43.5 (5–63)	56.66	0.005
Blinding of genotyping staff						
Blinded	6	1.07 (0.92–1.24)	0.391	62.6 (9–85)	13.36	0.020
Not reported	32	1.08 (1.00–1.16)	0.040	55.5 (34–70)	69.88	<0.001
Regenotyping of a random subsample						
Performed	5	0.94 (0.79–1.14)	0.544	58.9 (0–85)	9.74	0.045
Not reported	33	1.10 (1.03–1.18)	0.007	54.7 (33–69)	70.64	<0.001
Case definition						
>50%stenosis of ≥1 major vessels	16	1.09 (0.97–1.23)	0.135	62.4 (35–78)	39.9	<0.001
>70%stenosis of ≥1 major vessels	4	1.10 (0.90–1.34)	0.358	40.7 (0–80)	5.1	0.167
WHO criteria	14	1.00 (0.93–1.09)	0.942	36.9 (0–67)	20.6	0.081
Clinical diagnosis	4	1.31 (1.15–1.49)	<0.001	0.00 (0–85)	2.7	0.439
Source of controls						
Population-based	21	1.09 (1.01–1.19)	0.036	62.6 (40–77)	53.5	<0.001
Hospital-based	17	1.05 (0.95–1.17)	0.354	44.6 (2–69)	28.9	0.025

### Meta-regression

First, an empty regression was run with only the log of the effect estimate of pooled studies under the additive model to determine the baseline value for τ^2^, an estimate of between-study variation (baseline τ^2^ = 0.025). Next, single covariates were added in a series of univariate models. We performed the regression analysis for ten pre-defined potential sources of heterogeneity, including ethnicity, sex, mean age of cases, study size, case definition, source of controls, HWE-violation, blinding in genotyping, performing a sub-sample regenotyping, and matching (we hypothesized that studies that used matching might produce more conservative estimates of association). Univariate regression analyses showed that violation of HWE (β coefficient = 0.27 (0.06 to 0.48); *P_Het_* = 0.015, τ^2^ = 0.019), the mean age of cases (β = −0.01 (−0.02 to 0.0008); *P_Het_* = 0.066, τ^2^ = 0.024), and the method of case definition, clinically diagnosed CHD versus WHO criteria adjusted for other definitions (β = 0.26 (0.02 to 0.50); *P_Het_* = 0.038, τ^2^ = 0.020), were significant sources of heterogeneity among studies. The study size (*P_Het_* = 0.241, τ^2^ = 0.024), the ethnicity (*P_Het_* = 0.591, τ^2^ = 0.025), the male percentage in the study (*P_Het_* = 0.701, τ^2^ = 0.029), blinded genotyping (*P_Het_* = 0.890, τ^2^ = 0.026), sub-sample regenotyping (*P_Het_* = 0.131, τ^2^ = 0.023), the source of controls (*P_Het_* = 0.640, τ^2^ = 0.025), and matching (*P_Het_* = 0.942, τ^2^ = 0.026) were not significant sources of heterogeneity among studies. Violation of HWE in multivariable regression analysis remained a statistically significant source of heterogeneity after adjustment for the effect of study size (*P_Het_* = 0.031, τ^2^ = 0.020). Adding the mean age of cases and method of case definition to the model with violation of HWE decreased the τ^2^ value to 0.017 (*P_Het_* = 0.073 for violation of HWE, *P_Het_* = 0.057 for the mean age of cases, and *P_Het_* = 0.162 for clinically diagnosed CHD). It also showed that the effect of method of case definition on the variation among the studies was through the effect of the mean age on the heterogeneity and not as an independent factor. A model that included only violation of HWE and the mean age of cases reduced the τ^2^ value to 0.018 (*P_Het_* = 0.019, and 0.052, respectively).

### Sensitivity Analysis

First, the influence of deviation from the HWE on effect estimates was examined by using HWE-deviated adjusted ORs. Table 6 presents the genotype-based contrasts with corrected ORs, as well as the allele-based contrast. After adjustment, a smaller overall effect was seen under the additive, dominant, and pairwise comparisons. Moreover, after adjustment, the previously significant association under the additive model, as well as the TT vs. MM comparison, was no longer statistically significant. The association under the recessive model still remained significant.

**Table 6:** ORs and 95% CI after adjustment for HWE-deviation under different genetic models.

Genotype contrasts	Population	Number of studies	Random effects model			*I^2^* (%) (95%CI)	*Q* statistic for heterogeneity	*P*-value for heterogeneity
			Odds ratio	95%CI	P-value			
Additive	All	38	1.11	0.81–1.53	0.522	0 (0–37)	2.04	1.000
	Caucasians	25	1.11	0.75–1.64	0.616	0 (0–44)	1.04	1.000
	East Asians	8	1.19	0.60–2.36	0.626	0 (0–68)	0.82	0.997
Recessive	All	38	1.14	1.04–1.26	0.007	56 (37–70)	84.66	<0.001
	Caucasians	25	1.15	1.03–1.29	0.014	56 (32–72)	55.02	<0.001
	East Asians	8	1.18	0.90–1.55	0.242	73 (45–87)	26.15	<0.001
Dominant	All	38	1.05	0.96–1.15	0.330	49 (26–65)	72.52	<0.001
	Caucasians	25	1.08	0.98–1.20	0.121	58 (35–73)	57.82	<0.001
	East Asians	8	0.92	0.64–1.33	0.656	33 (0–70)	10.41	0.166
MT vs MM	All	38	1.00	0.92–1.09	0.996	15 (0–43)	43.41	0.217
	Caucasians	25	1.03	0.94–1.14	0.497	25 (0–54)	31.99	0.127
	East Asians	8	0.82	0.60–1.11	0.204	0 (0–68)	6.53	0.480
TT vs MM	All	38	1.13	0.99–1.28	0.080	52 (31–67)	77.88	<0.001
	Caucasians	25	1.19	1.02–1.38	0.023	60 (38–74)	60.11	<0.001
	East Asians	8	1.01	0.65–1.59	0.952	50 (0–77)	13.87	0.054

**Figure 3:**
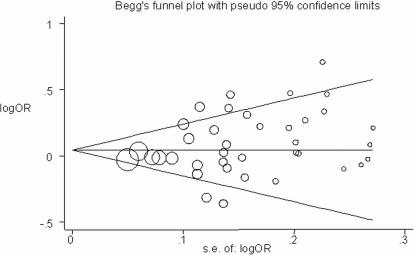
Begg's funnel plot with pseudo 95% confidence limits under the additive genetic model. The size of the circle is proportional to the weight of the study.

**Figure 4:**
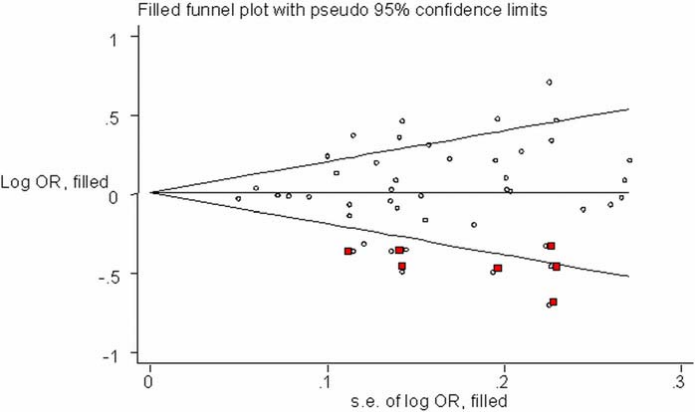
Filled Begg's funnel plot with pseudo 95% confidence limits under the additive genetic model. Red squares are missed studies due to publication bias.

Figure 3 shows a funnel plot in which the log of the OR of CHD risk under the additive genetic model was plotted against the standard error of the log of the OR in each study. The funnel plot for the overall results was substantially asymmetric for small negative studies. Moreover, tests for potential publication bias (The Egger's test and the Begg-Mazumdar test; *P*-values equal to 0.066 and 0.074, respectively) suggested the presence of a publication bias. By using the trim and fill method, we showed that, if the publication bias was the only source of the funnel plot asymmetry, it needed seven more studies to be symmetrical (Figure 4).

## Discussion

### Prospect-EPIC study

In this prospective study of healthy women aged 49 to 70 years, we investigated the relationship between the M235T polymorphism in the *AGT* gene and risk of AMI and CHD later in life. Under the additive genetic model, increased risks, albeit not statistically significant, were found for the incidence of AMI and CHD, which did not alter after adjustment. Likewise, we did not find a clear association between the variant and risk of CHD or AMI using different genetic models. This may be explained by: (i) the absence of a biological effect, (ii) the presence of real genetic heterogeneity according to ethnic background, or (iii) failure to detect a small effect because the epidemiologic risk for an individual genetic variant is likely to be small and a large sample size is needed for adequate statistical power. It has been commonly proposed that, as well as a need for much larger and more rigorous studies those that are currently used, there is a greater need for international collaborations, particularly for a complex disease like CHD [59].

#### Strengths and limitations

In our study, the data collection was prospective, before the diagnosis of AMI or CHD and equal for all participants. This ensures that the cases and the randomly selected controls are comparable [17]. For a multifactorial trait, like CHD, this provides a valid approach to evaluate the relationship between genetic factors and the risk of AMI and CHD, while taking into account co-existing and risk-modifying factors. In this study, prevalent cases of CHD were excluded from the analyses to prevent introducing bias due to potentially selective survival. The Prospect study was a population-based cohort, which makes it less susceptible to selection bias. Additional strengths were the comprehensiveness of our data and sample collection, as well as the morbidity and mortality follow-up for the entire cohort through linkage with nation-wide registries. The case-cohort design of the study combined the advantages of cohort studies (multiple outcomes and time-dependent covariates) with those of case-control analyses (fewer subjects); thus, it was more efficient than cohort studies. Classical case-control studies might be affected by selection bias since only non-fatal cases can be included, which was not the case in this study because of our endpoint definition. Moreover, we did not have misclassification of exposure (genotypes), which, when present, generally lead to a bias toward the null because we used standard laboratory protocols, performed a random double-check to detect potential genotyping errors, and had our *AGT* genotypes in Hardy-Weinberg equilibrium. The limitations of this study were the relatively short period of follow-up and the small number of cases. Moreover, because this cohort was exclusively composed of Dutch women, these results cannot be generalized to men or other ethnic groups, for whom the rates of the events or the allele frequency are known to differ.

### Meta-Analysis

The current meta-analysis, which includes new data from a prospective study in a large population-based cohort of Dutch women, represents a comprehensive evaluation of the M235T variant of the *AGT* gene in CHD risk. Although a pooled per-allele OR was suggestive of a modest increase in the risk of CHD of 1.08 (95% CI, 1.01 to 1.15), the robustness of this summary estimate is uncertain. First, in the pre-specified sub-groups analyses in the meta-analysis, larger studies, those with validated genotyping quality controls, and studies that used standardized criteria for case definition did not provide strong evidence for a positive statistically significant association between the M235T variant of the *AGT* gene and CHD risk. Second, the meta-regression analysis revealed that the HWE violation was a significant source of the moderate to large heterogeneity in the meta-analysis. Taking violation of HWE into account in the meta-analysis decreased the overall effect (Table 5). Third, the previous result was confirmed by using HWE-deviation adjusted ORs in the meta-analysis (Table 6). Moreover, there was evidence for publication bias in the meta-analysis. Taken together, these findings point to a violation of HWE and publication biases as the potential explanations for the results observed in the meta-analysis.

Some aspects of the current meta-analysis need to be considered to appreciate the findings. First, it might not be very practical to adjust for violation of HWE in the studies that mentioned that the violation is not due to genotyping errors. However, in the current meta-analysis, the HWE-violated studies that were included in the pooled estimate did not provide any reason for the violation. Therefore, we performed sensitivity analyses by using HWE-adjusted ORs and corresponding variances. Thereafter, a smaller overall effect was seen under most of the genetic models. Second, the power of tests for HWE and the power to detect genotyping errors are low. Therefore, the inability to detect a deviation from the HWE does not mean that there is no deviation, nor does it rule out the presence of genotyping errors, especially for small sample sizes. Third, our meta-analysis was based on published studies and we did not have access to the original data. However, it could be possible that an association between the genotype and disease exists in certain contexts rather than in all people studied. For example, a case-control study showed that the TT genotype was associated with an increased risk of CHD and MI only in smokers [33]. Finally, in all meta-analyses of gene-disease association studies, the inclusion criteria of cases and controls can be a potentially confounding factor. In this meta-analysis, cases were well defined and the source of controls was not a significant source of variation. However, the advantages of this study were the large sample size of the meta-analysis of 38 studies with 13284 cases and 18722 controls, which was twice the number of studies and sample sizes that had been reported in the previous meta-analysis [14], the exploration of potential sources of heterogeneity in the meta-analysis, and the evaluation of the association under different modes of inheritance.

Approximately 10% of gene-disease association studies are affected by statistically significant deviation from HWE, which could result from genotyping error, chance, inbreeding, non-random mating, differential survival of marker carriers, genetic drift, population stratification, or a combination of these reasons [20], [60]. Of these, genotyping error could be avoided by using standard genotyping methods and performing quality assessment. It has been recommended that authors specify the quality measures for the genotyping analysis, such as the blinding of laboratory staff to the donor subjects and hypotheses being investigated, procedures for establishing duplicates, degree of reproducibility between quality control replicates, and the inspection for conformity to HWE [61]. In the current meta-analysis, in studies where the blinding of genotyping staff was not reported, a statistically significant increased risk of CHD was found, while those that used blinding methods did not find a significant association. Moreover, for studies without regenotyping of a random sub-sample, a significant increase in CHD risk was found, but not for studies that performed regenotyping. Although overlapping confidence intervals for before-mentioned risks indicate caution in any interpretations, no report on blinding and regenotyping can point towards an uncertainty in quality control of genotyping in these studies. However, violation of HWE, which tends to inflate the chance of a false positive association, may be the strongest indicator of genotyping error [62].

Violation of HWE cannot solely explain the observed between-study variation in gene-disease association studies. The large between-study heterogeneity presented in most meta-analyses could be due to true heterogeneity (i.e., racial differences or differences in gene-environment interactions among various populations) or bias [63]. Bias, which could invalidate the results of the studies, should, therefore, be explored in detail. Biological plausibility, publication bias, selection bias, biased definition of cases, biased selection of controls, and population stratification should be assessed [63]. In this meta-analysis, we found strong evidence for publication bias. This is said to occur when the chance of the publication of a smaller study increases when it shows a stronger effect. Further exploration for sources of biases among studies showed that the selection of controls was not biased. However, using different case definitions resulted in a significant difference in the risk of CHD between those studies using WHO criteria and those using clinically diagnoses of CHD. Studies using definition of cases based on coronary angiography or based on WHO criteria had the same results. Considering a multivariate model in the meta-regression results, case definition was not a significant source of bias in the meta-analysis, while the different mean age of cases and violation of HWE were significant sources of heterogeneity. Since increasing age is a risk factor for CHD and the mean age of cases in the included studies ranged from 42 to 67 years, it is more likely that the studies with older individuals would show a stronger effect and produce heterogeneity. As case-parental controls, or other family-based designs, and genomic controls, using unlinked genetic markers which have no effect on the risk of CHD, were not available to evaluate the potential problem of population stratification among the studies, we presented effect estimates by different ethnic groups. However, there is controversy about the potential importance of population stratification for genetic-association studies using unrelated subjects [64].

In conclusion, the present meta-analysis, including our own data, indicated that, although a weak association between the M235T variant in the *AGT* gene and CHD was found, the relevance of this weakly positive overall association remains uncertain because it may be due to various residual biases. Moderate to large heterogeneity was identified between studies, and violation of HWE and the mean age of cases were statistically significant sources of the observed variation.
